# Depth accuracy of the RealSense F200: Low-cost 4D facial imaging

**DOI:** 10.1038/s41598-017-16608-7

**Published:** 2017-11-24

**Authors:** Timen C. ten Harkel, Caroline M. Speksnijder, Ferdinand van der Heijden, Carien H. G. Beurskens, Koen J. A. O. Ingels, Thomas J. J. Maal

**Affiliations:** 1 Radboud University Medical Centre, Radboudumc 3D Lab, Nijmegen, 6500 HB The Netherlands; 2 Radboud University Medical Centre, Department of Otorhinolaryngology and Head and Neck Surgery, Nijmegen, 6500 HB The Netherlands; 3 Radboud University Medical Centre, Department of Oral and Maxillofacial Surgery, Nijmegen, 6500 HB The Netherlands; 4 Radboud University Medical Centre, IQ healthcare, Nijmegen, 6500 HB The Netherlands; 5 University Medical Center Utrecht, Department of Oral and Maxillofacial Surgery, Utrecht, 3508 GA The Netherlands; 6 University of Twente, Robotics and Mechatronics, MIRA Institute for Biomedical Technology and Technical Medicine, Enschede, 7500 AE The Netherlands; 7 Radboud University Medical Centre, Department of Orthopedics, Section of Physical Therapy, Nijmegen, 6500 HB The Netherlands

## Abstract

The RealSense F200 represents a new generation of economically viable 4-dimensional imaging (4D) systems for home use. However, its 3D geometric (depth) accuracy has not been clinically tested. Therefore, this study determined the depth accuracy of the RealSense, in a cohort of patients with a unilateral facial palsy (n = 34), by using the clinically validated 3dMD system as a gold standard. The patients were simultaneously recorded with both systems, capturing six Sunnybrook poses. This study has shown that the RealSense depth accuracy was not affected by a facial palsy (1.48 ± 0.28 mm), compared to a healthy face (1.46 ± 0.26 mm). Furthermore, the Sunnybrook poses did not influence the RealSense depth accuracy (p = 0.76). However, the distance of the patients to the RealSense was shown to affect the accuracy of the system, where the highest depth accuracy of 1.07 mm was measured at a distance of 35 cm. Overall, this study has shown that the RealSense can provide reliable and accurate depth data when recording a range of facial movements. Therefore, when the portability, low-costs, and availability of the RealSense are taken into consideration, the camera is a viable option for 4D close range imaging in telehealth.

## Introduction

Three-dimensional (3D) and 4-dimensional (4D) imaging is extensively used in routine clinical practice, ranging from surgical planning and evaluation to patient monitoring, and rehabilitation^1–4^. A significant advantage of 4D imaging over 3D imaging, is that it can create multiple 3D images over time, which is especially suited for dynamic measurements, such as the movement of limbs or facial expressions^5^. Despite this, traditional 4D imaging systems tend to be bulky, expensive or overly complicated for self-patient use. Thus, their use has been limited to dedicated healthcare centers^1,6^. Technical developments have made it possible to create inexpensive, portable 4D cameras such as the RealSense F200 (which will be referred to as the RealSense). This may allow the shift of current 3D and 4D imaging tasks into telehealth applications^7^. However, before such an imaging device can be implemented in a clinical setting, it is crucial to evaluate the accuracy of the system.

**Figure 1:**
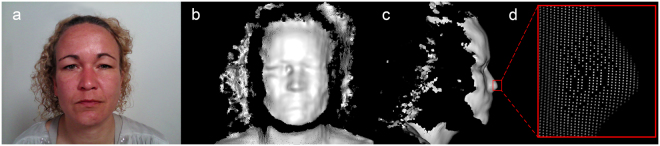
A single frame from a RealSense recording is shown, which simultaneously captures both the colour image (**a**) and the depth image (**b**), by the colour sensor and the IR sensor, respectively. During this study, the RealSense captured 27 of these frames per second. Although the recording was performed from a frontal position, it is possible to show the depth data from multiple angles, such as a lateral perspective (**c**), visualizing the additional available information. The individual points of the point cloud become visible when zooming in on the image (**d**). The colour frame was cropped and shading was added to the depth data for visualization purposes.

The RealSense is a portable 4D imaging device composed of five core elements: the image processor, colour sensor, infrared (IR) sensor, IR laser projector, and a stereo microphone. This device was developed for close range imaging, with a recommended user range of 20–120 cm, which allows the user to capture detailed areas such as the face or hand^8^. Typically, the RealSense will simultaneously capture colour and depth images, with a framerate around 30 frames per second (FPS). One single frame consists of a 2-dimensional (2D) colour image, captured by the light sensor (Fig. 1a) and a depth image, containing geometrical 3D information (Fig. 1b–d). The depth image is generated with the IR laser projector and the IR sensor. First, the IR laser projector emits a structured light pattern. Subsequently, the IR sensor captures the reflected light pattern from the object or person. The reflected pattern will be used to reconstruct the 3D surface, by a technique called triangulation^9^. The generated depth data consists of individual points with X, Y, Z coordinates resulting in a point cloud (Fig. 1d).

A possible telehealth application for the RealSense is the development of an automated scoring system for patients with a facial palsy, for monitoring rehabilitation progress at home. Currently, there exist over 19 subjective and objective scoring systems to grade the severity of a facial palsy^10–13^. One of the recommended subjective scoring systems is the Sunnybrook facial grading system. This scoring system is a well-established sensitive method for evaluating facial movement outcomes, both at rest and through five key voluntary movements (forehead wrinkle, gentle eye closure, open mouth smile, snarl, and lip pucker)^14^. Therefore, the Sunnybrook facial grading system is one of the most robust manual measuring methods currently in clinical use^10,11^. Thus, to incorporate the positive aspects of the Sunnybrook scoring system, and to make its clinical implementation easier, it would be valuable to create an automated scoring system based on the Sunnybrook scale. Since facial expressions consist of a significant amount of anterior-posterior movement^15^, a 4D system such as the RealSense could capture the information in this direction. However, currently there is no data available on the depth accuracy of the RealSense point cloud.

Therefore, the goal of this study was to determine the depth accuracy of the RealSense in a cohort of patients with a unilateral facial palsy capturing the face at rest with five additional voluntary movements based on the Sunnybrook scale. In addition, as this study was conducted in patients with a unilateral facial palsy, the unaffected side of the face of the patient was used to determine the depth accuracy of the RealSense in a healthy situation.

## Materials and Methods

### Population

In this study, patients presenting to the Radboud University Medical Centre (Radboudumc, Nijmegen, the Netherlands) with a unilateral facial palsy were included, irrespective of etiology, severity, and the time since onset of the palsy. The exclusion criteria were the presence of a bilateral facial palsy and an age <18 years. Approval of this study was authorized by the Ethics Committee of the Radboudumc (2015-1829). This study was conducted in compliance with the World Medical Association Declaration of Helsinki on medical research ethics. All subjects provided written informed consent before data acquisition. Additionally, a written informed consent was obtained from the patient shown in this paper, to publish the images in an online open-access publication.

### Data acquisition

**Figure 2:**
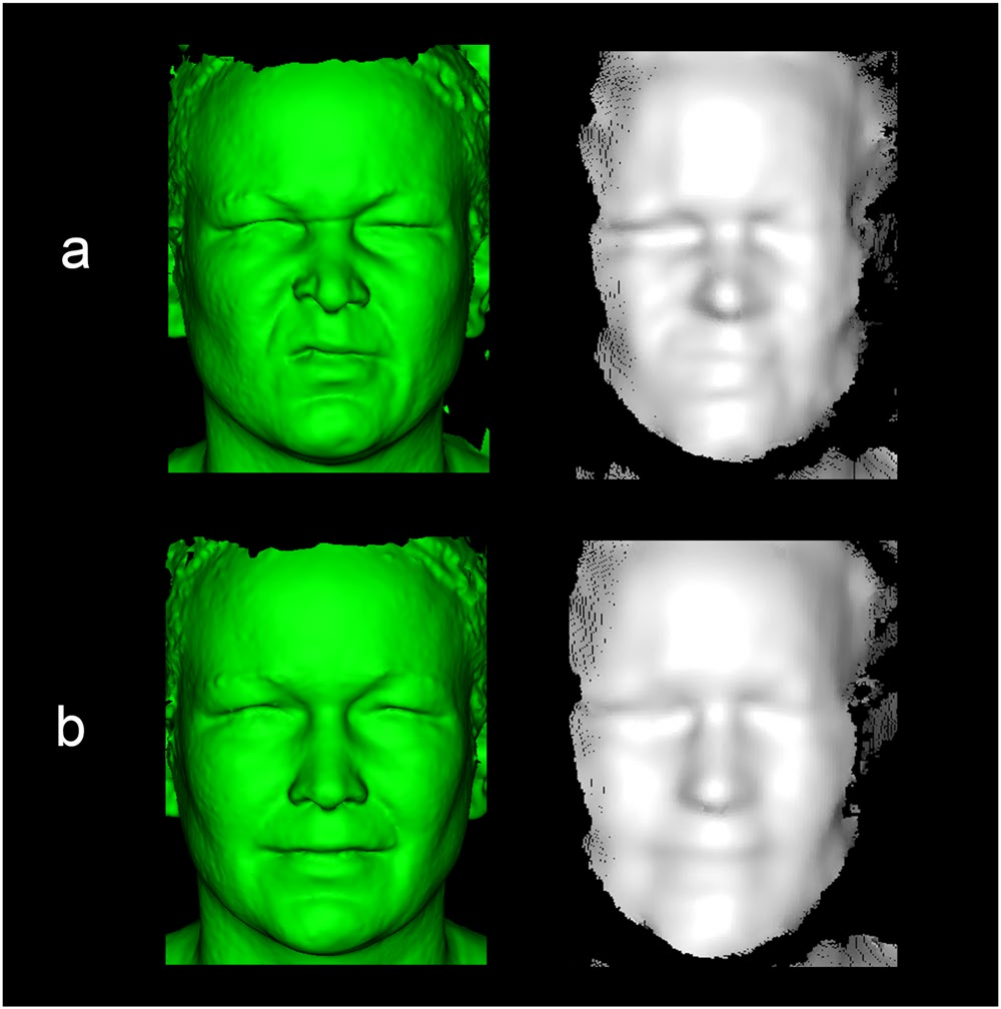
Comparison of the depth data between the simultaneously captured 3D reference image (3dMD system; green) and the RealSense depth image (white). A total of six Sunnybrook poses were captured for each patient with a unilateral facial palsy (n = 34), where the snarl (**a**) and smile (**b**) are shown as an example for a single patient. The 3D reference image acted as the gold standard, to determine the depth accuracy obtained by the RealSense.

Continuous RealSense recordings were acquired with the RealSense F200 (depth camera manager version 1.4.27 and RealSense Software Development Kit, RSSDK, version 7.0.23.8048, Intel, Santa Clara, USA) using a colour resolution of 1920 × 1080 pixels and depth resolution of 640 × 480 pixels. RealSense recordings were captured with an average frame rate of 27 FPS. Simultaneously, a two-pod 3dMD system (3dMDface, 3dMD, Atlanta, USA) was used to capture single static 3D images, acting as the reference clinical standard (Fig. 2). Patients were first positioned in front of the 3dMD camera. Subsequently, the RealSense camera was positioned in front of the patient at eye level on a tripod. The minimum distance from camera to patient was determined by the RealSense facial tracking algorithm from the RSSDK. The distance between patient and camera was increased if this was required due to physical limitations, such as body size. All recordings were acquired in a windowless room used for clinical 3D imaging at the department of oral and maxillofacial surgery. A diffuse lighting environment was created with two Diva Light 400 lights (Kinoflo Lighting Systems, Los Angeles, USA), which was the only light source in the room. Finally, a single RealSense recording was made for each patient, capturing six different poses based on the Sunnybrook facial grading system, which includes the face at rest and five facial expressions based on voluntary movements (forehead wrinkle, gentle eye closure, open mouth smile, snarl, and lip pucker)^14^. The patient was asked to hold each pose at maximum exertion of the voluntary movement, until the static 3D image was taken. A total of six static 3D images were captured with the 3dMD system during a single RealSense recording. The static 3D images made by the 3dMD system will be referred to as the 3D reference images.

### Data processing

To determine the accuracy of the RealSense, the RealSense depth data was compared to the 3D reference images for each Sunnybrook pose. Since the RealSense consisted of a continuous data stream, six frames from the RealSense recording were selected at the capture time of the 3D reference image. The frame selection was based on the flash from the 3dMD system that was visible on the RealSense recording. To prevent RealSense depth data distortion due to the 3dMD flash, the RealSense frame immediately prior to the 3dMD flash was used in the final analysis. From the six selected RealSense frames, which captured the facial movements at maximum exertion, the depth data was exported with the RSSDK as individual point clouds in X, Y, Z coordinates (Fig. 2).

**Figure 3:**
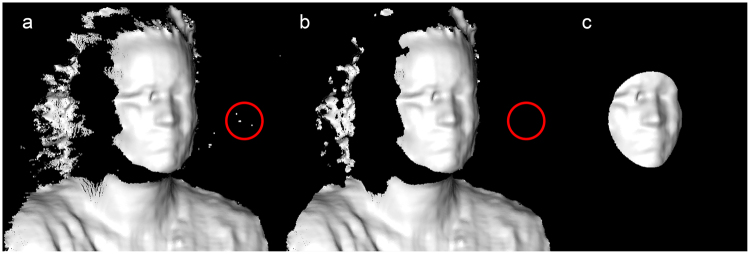
Overview of the pre-processing of RealSense depth images. The original image (**a**) showing the raw depth data. As exemplified in the red circle, this image still contains spurious background noise. Using a statistical outlier filter, the background noise was removed from the original RealSense depth data (**b**). After correcting for statistical outliers, a region of interest (ROI) was selected based on a sphere centred at the pronasale (**c**). The radius of the sphere was determined by the distance between the pronasale and the exocanthion, with an additional margin of 10%, to include the complete eye region.

**Figure 4:**
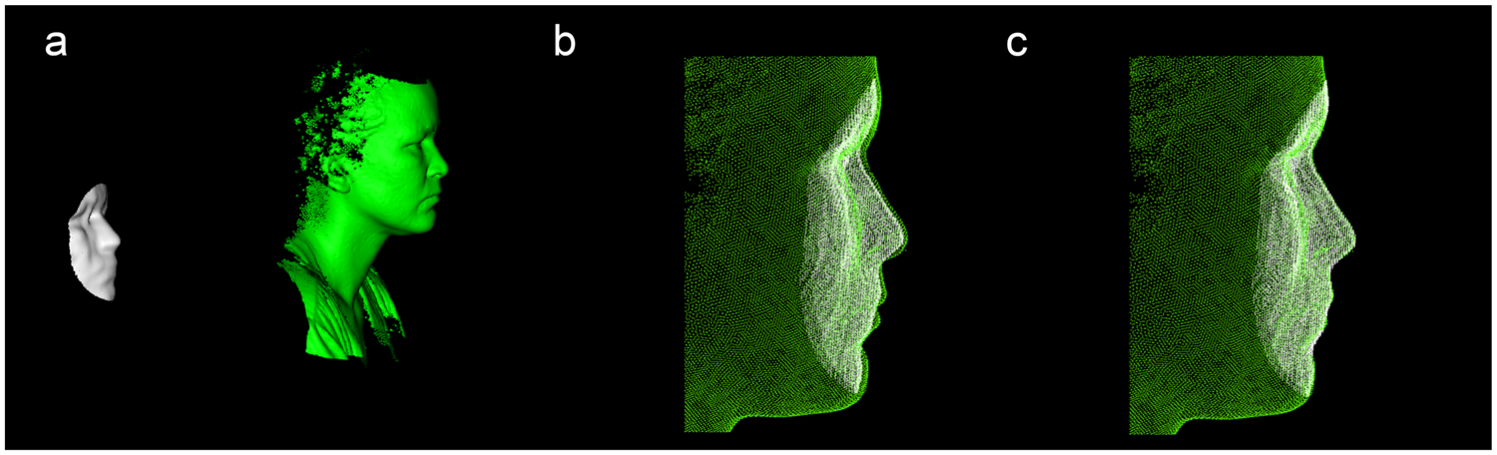
A registration pipeline was used to align the cropped RealSense image (white) with the 3D reference image (green). A rough alignment was performed with a Procrustes analysis (**b**). Subsequently a refined alignment was performed with the Iterative Closest Point Algorithm (**c**). The 3D reference image was cropped in subfigure b & c for clarity.

After exporting the point clouds, the pre-processing was performed using the Point Cloud Library (PCL, version 1.8.0)^16^. Due to a limited field of view of the RealSense depth image compared to the 3D reference image (Fig. 2), a region of interest (ROI) was selected from the RealSense image. To remove possible noise within the ROI, a statistical outlier filter was applied (Fig. 3b)^16^. Next, the ROI was selected with a sphere centred at the pronasale (Fig. 3c). The radius of the sphere was determined by the maximum Euclidean distance between the pronasale and the left or right exocanthion, based on manual landmarks placed on the 3D reference image. The sphere radius was increased by 10% to include the eye region completely. No pre-processing was applied to the 3D reference image. After the pre-processing stage, initial registration was performed between the RealSense point cloud and 3D reference image by the Procrustes algorithm implemented by libigl (Fig. 4a,b)^17^. The Procrustes algorithm was performed with manually placed landmarks, at the exocanthion and pronasale, at the RealSense and 3D reference image. During this registration no scaling or reflection was applied. The initial registration was followed by a refined registration with the Iterative Closest Point (ICP) algorithm implemented by PCL (Fig. 4c)^18^, set to a rigid registration without scaling, as not to deform the RealSense point cloud.

### Data analysis

**Figure 5:**
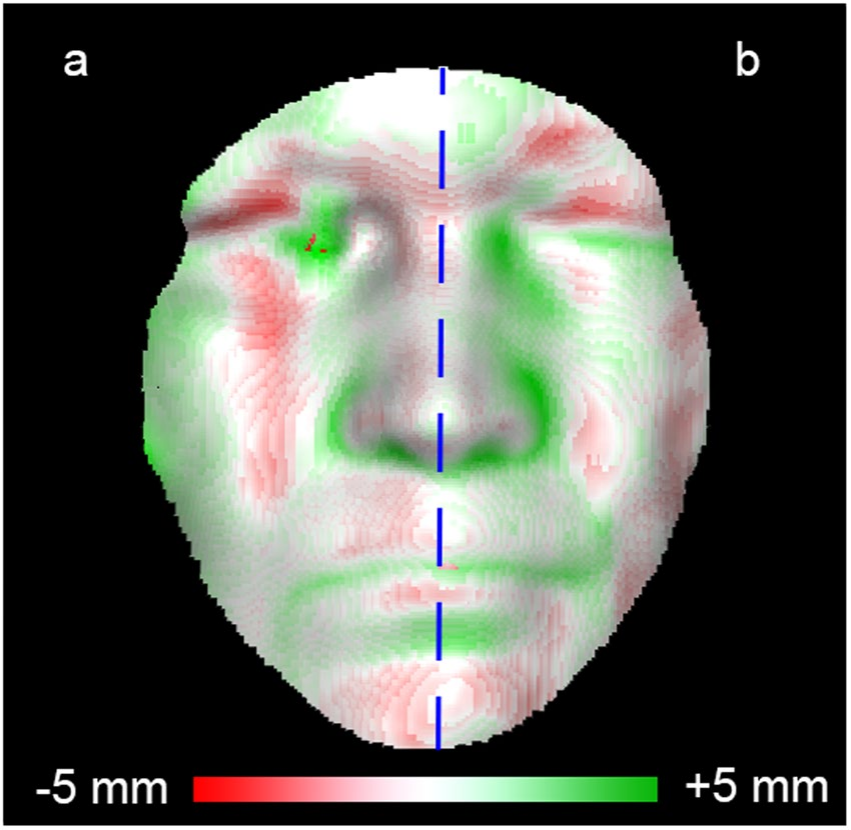
A distance map generated from a patient in rest. The distance map was created by calculating the closest distance between the RealSense image and the 3D reference image, for each RealSense point. The white areas represent a perfect match with the 3D reference image (0 mm), with areas in red and green showing distances between ±5 mm. Using the midsagittal plane (blue line), the distance map was calculated separately for the healthy (**a**) and palsy side (**b**) of the face.

To determine the accuracy of the RealSense point cloud, a distance map was calculated between the RealSense and the 3D reference image with PCL (Fig. 5). The distance map was created by calculating the Euclidian distance between each point of the RealSense point cloud to the closest point on the 3D reference image. The final depth accuracy was defined as the root mean square (RMS) of the distance map, where the 3D reference image was considered as the gold standard. This analysis was performed separately for the healthy and palsy side of the patient, determined by the midsagittal plane (Fig. 5), and each of the six Sunnybrook poses (rest, forehead wrinkle, gentle eye closure, open mouth smile, snarl, and lip pucker)^14^. A paired Student’s t-test was performed for each pose comparing the depth accuracy of the healthy and palsy side. Additionally, a one-way analysis of covariance (ANCOVA) was performed to determine if there were significant differences in depth accuracy between the Sunnybrook poses. In this analysis, the Sunnybrook poses were categorized as six different groups, with the depth accuracy acting as the dependent variable, and the RealSense camera distance as the covariate. The data from the paired Student’s t-test and the ANCOVA analysis was tested for normality using the Kolmogorov-Smirnov test with Lilliefors significance correction^19^. Additionally, the homogeneity of variances was tested with Levene’s test for the ANCOVA analysis^20^. A p-value of <0.05 was considered as statistically significant. Statistical analysis was performed using IBM SPSS Statistics, Version 22 (IBM Corp., Armonk, NY, USA).

## Results

A total of 34 patients were included in this study (age: 53 ± 13 years, gender: 71% female, left sided palsy: 53%). Each patient was captured with the face at rest and with five additional voluntary movements based on the Sunnybrook facial grading scale (forehead wrinkle, gentle eye closure, open mouth smile, snarl, and lip pucker), where the patient was simultaneously recorded with the RealSense and the 3dMD system. This resulted in the comparison of 204 RealSense point clouds with their associated 3D reference image (Fig. 2).

**Figure 6:**
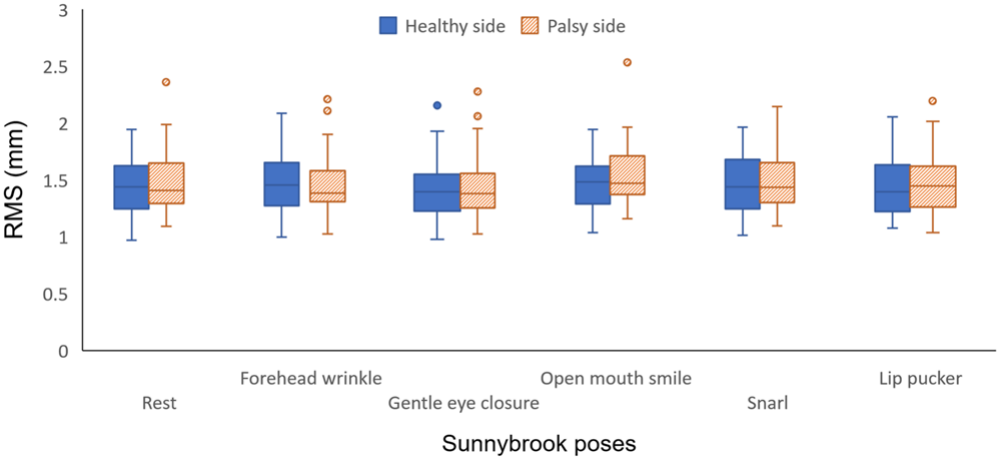
Average accuracy RealSense depth data (n = 34 for each Sunnybrook pose) comparing the healthy and palsy side of the patient. Depth accuracy is expressed as the root mean square (RMS). Any values greater than 1.5 times the interquartile range were considered as outliers for the boxplot.

Firstly, the depth accuracy for the healthy side of the face was calculated for all Sunnybrook poses combined, which lead to an average RMS of 1.48 mm (standard deviation (SD) = 0.28 mm; 95^th^ percentile (p95) = 2.08 mm). The palsy side of the face resulted in an RMS of 1.46 mm (SD = 0.26, and p95 = 1.93). The depth accuracies of the healthy and palsy side of the separate poses are shown in Fig. 6. No significant results were found in the Kolmogorov-Smirnov test for any of the exercises. Therefore, the data was assumed to be normally distributed. The paired Student’s t-test showed no statistically significant differences between the accuracy of the healthy and palsy side for any of the six poses (rest, p = 0.25; forehead wrinkle, p = 0.96; eye closure, p = 0.63; smile, p = 0.22; snarl, p = 0.41; lip pucker, p = 0.63).

**Figure 7:**
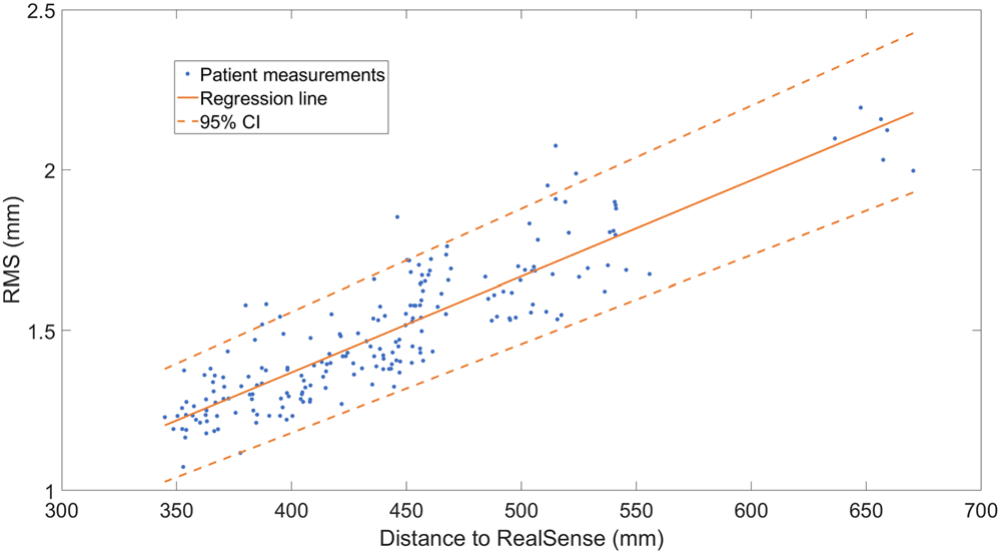
Correlation between the distance from patient to the RealSense camera and the depth accuracy of the RealSense. The patient measurements include all the six Sunnybrook poses for the 34 patients (n = 204). Depth accuracy is expressed as the root mean square (RMS) in mm. The regression line from the ANCOVA analysis is shown (y = 0.003x + 0.1715 with r = 0.78 and p = 0.00), including the 95% confidence interval (CI).

As we have shown that there is no significant difference in accuracy between the two sides of the face, the data from the healthy and palsy side of the face were combined in order to determine the depth accuracy in between the Sunnybrook poses (see Table 1). No significant results were found in either the Kolmogorov-Smirnov test or Levine’s test in the data of the ANCOVA analysis. The ANCOVA analysis showed no significant differences in depth accuracy between the poses (F = 0.53, p = 0.76). When combining the data from the six poses with the ANCOVA analysis, an average linear regression of y = 0.003 x + 0.1715 (r = 0.78, p = 0.00) was found as shown in Fig. 7, where x is the distance to the camera in mm and y the depth accuracy in mm.

**Table 1:** Depth accuracy of the RealSense depth data in patients with a unilateral facial palsy grouped by the six Sunnybrook poses with the healthy and palsy side combined (n = 34 for each pose). Depth accuracy is expressed as the root mean square (RMS).

Sunnybrook pose	RMS ± SD (mm)	p95 (mm)
Rest	1.48 ± 0.22	1.95
Forehead wrinkle	1.49 ± 0.20	1.90
Gentle eye closure	1.46 ± 0.24	1.89
Open mouth smile	1.53 ± 0.22	2.04
Snarl	1.49 ± 0.22	1.93
Lip pucker	1.48 ± 0.22	1.83

## Discussion

In this study, the depth accuracy of the RealSense was determined in a cohort of 34 patients with a unilateral facial palsy. The patients were recorded with the face at rest with five additional voluntary movements (forehead wrinkle, gentle eye closure, open mouth smile, snarl, and lip pucker), which were based on the Sunnybrook facial grading system^14^. No significant differences were found in depth accuracy when comparing the healthy and palsy side of the patients, with an average RMS of 1.48 ± 0.28 mm and 1.46 ± 0.26 mm respectively. Additionally, no significant differences were found in depth accuracy between the Sunnybrook poses (p = 0.76).

To the best of our knowledge, this is the first study investigating the depth accuracy of the RealSense. Therefore, no direct comparison can be made with other studies investigating the depth accuracy of the RealSense. Although other “off-the-shelf” 4D imaging systems such as the Kinect (Version 1 and 2, Microsoft, USA), have been used to reconstruct a face using multiple frames (RMS accuracy 0.84–2.0 mm^21–25^). No comparable studies have used a single frame of the face and compared this to a clinical reference standard, such as the 3dMD system. Therefore, our study has shown that the RealSense depth accuracy lies within the range of the Kinect accuracy when imaging the face, but it is expected to be higher due to use of only a single frame for the analysis of the RealSense.

The depth accuracy of the RealSense camera was determined by comparing the RealSense data to the 3D reference image from the two-pod 3dMD system. The reference image has a known accuracy of 0.2–0.25 mm when imaging the face at rest, which is considered as a sufficient accuracy for a range of clinical implementations^26–29^. Therefore, the RealSense would have a similar accuracy as the 3dMD system if the depth accuracy of the RealSense had been in the range of 0.25 mm. However, the RealSense is an order of magnitude more inaccurate, with an average accuracy of 1.48 mm for the healthy face at rest. This resulted in a smoother RealSense image compared to the 3D reference image (Fig. 2). Therefore, a decrease in accuracy at regions with a higher curvature, such as the mouth and nose, is expected and can be seen in Fig. 5. The inaccuracies around the nose region can partially be explained by the blocked view around the alar groove due to the frontal positioning of the RealSense, whereas the 3D reference image was captured by two pods from the side.

The accuracy of the RealSense was shown to decrease when imaging the eye region. However, it is known that the accuracy of the 3D reference image decreases when capturing specular surfaces, such as the eye and teeth^30^. This inaccuracy in the gold standard was not corrected for during this study, since the average accuracy of the 3D reference image is 0.38 ± 0.34 mm around the eye^31^, which is still an order of magnitude more accurate than the RealSense. The impact on the depth accuracy can for example be seen between the neutral pose, and the gentle eye closure, where the specular area of the eye is covered, which resulted in a non-significant difference in the average accuracy in this study (Table 1). Overall, the apparent difference in depth accuracy between the RealSense image and the 3D reference image was an expected result, considering the difference in cost, size, and complexity of the two systems.

Further analysis compared the accuracy between the healthy and palsy side for the RealSense. A possible difference in accuracy, between the healthy and palsy side, could have been found due to the asymmetrical nature of the face in palsy patients. For example, palsy patients can experience a dropped corner of the mouth, or a pronounced labial fold, in the affected side of the face at rest^14^. This can lead to an increased complexity of the facial surface. However, no significant differences in depth accuracy were found in this study for any of the Sunnybrook poses when comparing the healthy side to the palsy side. This indicates that the RealSense is able to capture the depth information of the asymmetrical features of the patients for all the Sunnybrook poses. Although this study found that the RealSense has an average accuracy ranging between 1.46 mm and 1.53 mm, the average facial movement is expected to be 6.49 mm in the vertical direction and 5.49 mm in anterior-posterior direction in a healthy situation^32^. Therefore, the surface differences between the healthy and palsy side of the face seem to be large enough to be detected by the RealSense.

An important consideration when analysing the depth accuracy of the RealSense is the influence of patient to camera distance. Cameras that acquire depth data with structured light patterns, such as the RealSense, are expected to increase their depth accuracy at closer distances^33^. During this study, the minimal camera distance to the patient was determined by a facial tracking algorithm built in with the software development kit of the camera, with an operating range of 30–100 cm^8^. However, since the patients needed to be captured simultaneously with the 3dMD system, the available imaging space was limited. Due to physical limitations, such as body size, the camera distance needed adjustments for each patient to make the recording possible. This resulted in the majority of patients being recorded in a range of 35–55 cm, with one patient being measured at a distance of 65 cm. However, when the healthy side of the face was compared to the palsy side, the distance to the camera was approximately the same since the patients were positioned perpendicular to the RealSense. Therefore, the intra-patient accuracy was minimally influenced. In contrast, the inter-patient accuracy is heavily influenced by the distance to the camera, as can be seen in Fig. 7 (r = 0.78, p = 0.00). Therefore, the average RMS, SD, and p95 reported in this study highly depend on the distance to the camera and the selected distance range. This will represent a realistic scenario for certain real-world clinical implementations where the distance to the camera will vary between measurements. For example, in this study patients moved in between the captured Sunnybrook poses, as can be seen in Fig. 7 at the patient measured at 65 cm. Therefore, an ANCOVA analysis was applied to correct for the distance to the camera, when comparing depth accuracy between the six poses. Since no significant differences were found (p = 0.76), the RealSense was tested in a wide range of facial motion, without showing significant differences in depth accuracy.

The current study design has several limitations that should be taken into account. First of all, the cohort consisted of unilateral facial palsy patients ≥18 years, making the depth accuracy unknown for children, healthy adults, and other diseases. Additionally, the accuracy of structured light cameras is known to be influenced by different light sources^33^, which was not investigated in this study. When applying the RealSense in telehealth applications more various lighting conditions can be expected. Therefore, future research should determine the influence of the lighting in the room on the accuracy of the RealSense. The current measurement setup used a single RealSense camera, compared to the two pods of the 3dMD system. This resulted in a more limited field of view for the RealSense (Fig. 2), possibly losing valuable information of the face. To overcome this limitation, it is possible to use multiple synchronized RealSense cameras, positioned at different angles. However, this will increase the complexity of the measurement setup that needs to be used at home. Therefore, this study used a single RealSense camera, and an ROI was selected to make the comparison between the RealSense and the 3D reference image possible. The ROI included key areas of the face, such as the eyes and mouth. To make the ROI consistent for all patients, the area around the pronasale was selected within a patient specific radius (Fig. 3c). This radius was determined by the distance between the pronasale and the exocanthion, to include the eye region. To prevent cropping of the eye region, 10% was added to the determined radius. The ROI used in this study was relatively conservative, to make sure a similar ROI could be selected in between patients. The average depth accuracy potentially could have improved, since the excluded areas immediate to the current ROI were areas with low curvatures. However, the point cloud was cut off at the lateral sides of the face (Fig. 1c), since these areas were positioned more perpendicular to the camera. The exact position of this cut-off changed in between patients. Therefore, a conservative ROI was selected to select the same ROI in between patients. This ROI can be increased by using multiple synchronized RealSense cameras positioned at different angles in the measurement setup.

Additionally, during the processing of the data it was necessary to apply a registration between the RealSense and the 3D reference image, since the two images were captured with two separate imaging devices, resulting in a different location in space (Fig. 4a). With the implementation of the Procrustes and ICP registration, it was possible to match the point clouds semi-automatically. However, the final ICP registration could find a sub-optimal matching in a local optimum, resulting in a lower depth accuracy^34^. In addition, another important limitation to this study, is that the clinical reference standard was only able to capture static 3D images. Therefore, only a single frame of the RealSense recording could be used in the final analysis for each Sunnybrook pose, while there are 27 RealSense frames available each second. Future studies would benefit from the use of a professional 4D system as the reference clinical standard. However, in this study, six frames were extracted from each RealSense recording, capturing the accuracy of the system over multiple time points. All six Sunnybrook poses reported an accuracy within a range of 1.46–1.53 mm, showing the consistency of the camera for various facial movements over time, in a single recording.

In conclusion, this study has shown that the RealSense can provide reliable and accurate depth data when capturing the face at rest and when performing five voluntary movements based on the Sunnybrook facial grading system, in a cohort of 34 patients with a unilateral facial palsy. Therefore, a similar accuracy of the RealSense point cloud can be expected when analysing the different Sunnybrook poses, when an automated Sunnybrook scoring system is implemented. Additionally, it has been shown submillimetre information is lost in the RealSense point cloud, especially noticeable in areas with higher curvature, which will need to be taken into account in an automated scoring system. However, larger deviations will be possible to capture, especially at a closer distance to the camera, where the highest depth accuracy of 1.07 mm was achieved at a distance of 35 cm. Due to the correlation between camera distance and depth accuracy for systems such as the RealSense^33^, it will be essential to keep track of the patient to camera distance in clinical applications. One aspect that needs to be included in future research is the influence of the lighting in the room on the accuracy of the RealSense. Although this study investigated the imaging of facial palsy patients with the RealSense, there are numerous applications for a portable 3D and 4D imaging system such as the RealSense. With the emerging interest in the use of telehealth in tasks such as health monitoring, diagnostics, and performing consults, there is still room to increase the use of 3D and 4D imaging in telehealth^35–42^. Overall, when considering the portability, low-costs, and availability of the RealSense, the camera is a viable option for 3D and 4D imaging in telehealth, where the RealSense is especially suited for close range imaging. However, when submillimetre accuracy is required for the clinical application, more professional setups are still recommended to be used.
